# Translating the Cluster Headache Quality of Life Questionnaire (CHQ) from English to Dutch with the TRAPD method

**DOI:** 10.1007/s10072-023-07088-x

**Published:** 2023-10-06

**Authors:** Willemijn C. Naber, Roemer B. Brandt, Dimitris D. Figetakis, Marjan Jahanshahi, Gisela M. Terwindt, Rolf Fronczek

**Affiliations:** 1 Department of Neurology, Leiden University Medical Centre (LUMC), K5-Q-93, Albinusdreef 2, 2333 ZA Leiden, The Netherlands; 2 Cognitive Motor Neuroscience Group, Department of Clinical and Motor Neurosciences, UCL Queen Square Institute of Neurology and The National Hospital for Neurology and Neurosurgery, Queen Square, London, WC1N 3BG UK; 3 Stichting Epilepsie Instellingen Nederland (SEIN), Sleep-Wake Centre, Heemstede, The Netherlands

**Keywords:** Cluster headache, Trigeminal autonomic cephalalgia, Quality of life, Patient-reported outcome measurement, Cluster Headache Quality of Life Questionnaire

## Abstract

**Objective:**

Cluster headache is associated with a decreased quality of life (QoL). The increased focus on patient-reported outcome measures (PROMS) has led to the creation of a tailored Cluster Headache Quality of Life scale (CHQ). Our objective was to create and authenticate a Dutch version of the CHQ (CHQ-D).

**Methods:**

The TRAPD model (Translation, Review, Adjudication, Pretesting, Documentation) was used to translate the CHQ from English to Dutch and ensure cross-cultural adaption. Pre-testing was performed in *n* = 31 participants, and validity was in a new sample of *n* = 40 participants who completed the CHQ twice at a 2-day interval. Intraclass correlation coefficient (ICC) and Cronbach’s alpha were used to assess the validity and reproducibility of the CHQ-D.

**Results:**

To produce the CHQ-D, we made five modifications based on pretesting. Participants finished the questionnaire in a median time of 10 min (IQR:10.0, 17.5) and 90% within 20 min. The majority of participants (74.2%) did not find it burdensome at all. The reliability of the CHQ-D was excellent (Cronbach’s alpha: 0.94; ICC: 0.94).

**Conclusion:**

The CHQ-D is a valid and practical instrument for QoL in individuals with cluster headache. We aim to use CHQ-D as PROM in clinical research in the Netherlands to enforce international collaborations and comparisons of studies.

**Supplementary information:**

The online version contains supplementary material available at 10.1007/s10072-023-07088-x.

## Introduction

Cluster headache is associated with a decreased quality of life (QoL) [1]. In recent years, there has been greater emphasis on patient-reported outcome measures (PROMs). In line with this development, the newly revised clinical trial guidelines for cluster headache advised to incorporate these measures as clinical trial end point [2]. Despite the specific characteristics of cluster headache, no validated QoL questionnaire for cluster headache was available until 2016, when the English version of the Cluster Headache Quality of Life scale (CHQ) was developed [3].

The CHQ is a short, easy-to-use questionnaire relating to patients’ day-to-day lives. The scale was developed in consultation with people with cluster headache and clinicians. The CHQ consists of 28 questions that include four domains related to QoL: “restriction of activities of daily living,” “impact on mood and interpersonal relationships,” “pain and anxiety,” and “lack of vitality” [3]. The English CHQ questionnaire is validated and reliable [3].

The CHQ is not available in Dutch (or any other foreign language other than English), leading to the usage of the, less ideal, generic QoL questionnaires such as the Short-Form-36 in the Netherlands [4]. The use of a foreign questionnaire is prone to bias with linguistic nuances and cultural aspects that might lead to an incorrect interpretation of the outcome. This study therefore aimed to develop and validate a Dutch translation of the CHQ [5]. To ensure correct interpretation and accurate results, adequate translation methods need to be used. An established method for doing this is the TRAPD (Translation, Review, Adjudication, Pretesting, and Documentation) team translation model. Here we report on the translation and validation of the Cluster Headache Quality of Life scale from English to Dutch using this TRAPD model.

## Material and method

### Study design

**Fig. 1:**
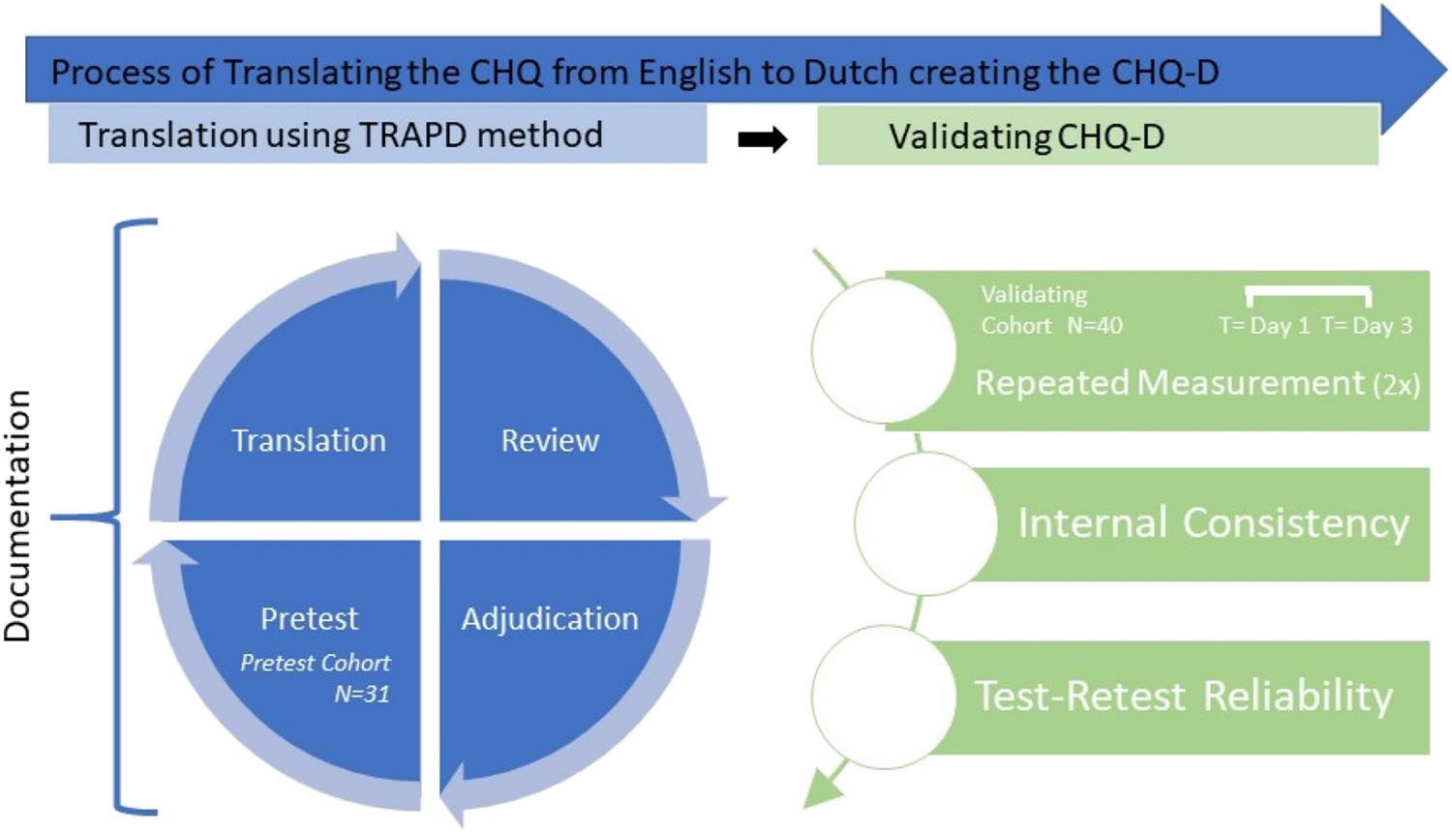
Overview of the process of translating and validating the CHQ, resulting in the CHQ-D. Left side: The CHQ was translated using the TRAPD method (5 steps: *Translation, Review, Adjudication, Pretest, Documentation*) including preliminary testing of the questionnaire in a pretest cohort of 31 participants with cluster headache. Right side: The CHQ-D was validated using a validation cohort of 40 participants with cluster headache who completed the questionnaire twice with a 2-day interval after with the internal consistency, and test-retest reliability was calculated. Legend: CHQ, Cluster Headache Quality of Life Questionnaire; CHQ-D, Cluster Headache Quality of Life scale—Dutch version

To translate the CHQ from English to Dutch and ensure cross-cultural adaption, a multi-step and team-based translation process in conformity with the TRAPD model (Fig. 1) was used [5]. After the definite Dutch translation was achieved, the scale was validated in the second part of this project. Informed consent was obtained from all participants. As the current study only involved a non-burdensome questionnaire, the provided informed consent was sufficient for study participation according to the Dutch law for medical research (Medical Research Involving Human Subjects Act). Therefore, an exemption for additional medical ethical review was provided by the Medical Research Ethics Committee of the LUMC ((METC-LDD; Reference number 22–3008). Data were collected between June 2022 and October 2022.

#### Cluster Headache Quality of Life scale (CHQ)

The original CHQ was provided by the designers of the questionnaire (Abu Bakar et al. [3], supplementary file 2). The CHQ scale consists of 28 items, in which the frequency of certain activities and emotions is scored. Each item is answered using a 5-point Likert scale (“never” (= 0), “occasionally” (= 1), “sometimes” (= 2), “often” (= 3), “always” (= 4)). The minimum obtainable score of the total questionnaire is 0, the maximum is 112. Higher scores indicate a poorer health-related QoL. In addition to the total score, four subscores can be calculated corresponding to four subdomains: (i) “restriction of activities of daily living” (items 1–9), (ii) “impact on mood and interpersonal relationships” (10–21), (iii) “pain and anxiety” (22 and 23), and (iv) “lack of vitality” (24–28). Lastly, a 100-mm visual analogue scale, ranging from “not at all satisfied” to “very satisfied,” is included at the bottom of the original questionnaire. This scale is scored according to the distance from the left side of the scale to the drawn line of the patient. Higher scores (i.e., more distance from the left side) indicate better overall health-related QoL. This score is not included in the total CHQ score and is reported separately.

### Participants

Participants were selected from the Leiden University Cluster headache Neuro Analysis (LUCA) cohort [6]. The LUCA cohort is a validated, web-based cohort with a screening questionnaire for cluster headache based on the ICHD-3 criteria [6]. Patients participated either in the translation process (*n* = 31) or in the validation process (*n* = 40). Inclusion criteria were as follows: being a native-Dutch speaker, being 18 years or older, and having a diagnosis of episodic or chronic cluster headache as defined by the ICHD-3 criteria [7]. Participants who were attack-free for > 3 years were excluded. Sociodemographic data about participants including age, sex, level of school education, and disease-specific information (type of cluster headache, attack frequency) were collected.

#### Translation process

The translation was performed with the use of the TRAPD method. This method was originally developed by Janet Harkness and is the preferred method for the translation and adaption of questionnaires according to the Cross-Cultural Survey Guidelines [5, 8]. This method consists of 5 different steps: (i) Translation, (ii) Review, (iii) Adjudication, (iv) Pretest, (v) Documentation (Fig. 1). All steps of the translation process (different translated versions, discussion notes, etc.) were carefully documented.

(i) Two translators (RH and DF) both proficient in English and native Dutch speakers with experience in the cluster headache field independently translated the questionnaire from English to Dutch.
(ii) The two preliminary translations were reviewed by the translators and an independent reviewer (WN). For each question, the best wording was discussed to achieve a single pre-final translation.
(iii) The pre-final Dutch translation was compared and considered equal to the original (English) version by the adjudicator (RB). This pre-final translation was used for the *Pretest*.
(iv) The pre-final questionnaire was pretested in the “pretest cohort.” During the pretest, participants were asked to complete the questionnaire online. Furthermore, participants were offered the possibility to leave remarks about clarity and wording after each question. Lastly, survey burden was evaluated using a 6-point Likert scale (1, “not burdensome at all”–6, “very burdensome”). An interview by phone was conducted with all participants when they had completed the questionnaire. Their interpretation of each of the questions and any perceived ambiguities were evaluated. Finally, participants were asked if they had any additional comments or remarks.
(v) All feedback that was collected during the pretest was reviewed by the two individual translators and the reviewer by repeating the first three steps of the TRAPD model until an agreement was reached on the revised final version of the translation. Hereafter, the final translated Dutch version of the CHQ will be called the CHQ-D (“Cluster Headache Quality of Life scale – Dutch version”).

#### Validation of the CHQ-D

The reliability and validity of the CHQ-D were tested in a new sample of 40 participants (Fig. 1), who were instructed to complete the questionnaire twice at a 2-day interval. Due to the inherent fluctuations in disease activity and possible confounding factors, the retest interval should be as short as possible, while avoiding recall. A 2-day interval was shown to be equivalent to a 2-week interval [9]. Participants were asked to complete the questionnaire both times in a comparable setting (e.g., at home in the evening).

### Statistical analysis

Descriptive data are presented as number (percentage) or median (interquartile range)/mean (SD), depending on the distribution of the data. For group comparisons, chi-square tests, Fisher’s exact tests, Student’s *t*-tests, or Mann–Whitney tests were performed when appropriate. A chi-square test was used to assess if the level of education was associated with the number of remarks on the questionnaire during the preliminary test.

The floor effect was quantified as the percentage of patients who achieved the minimal score and the ceiling effect as the percentage achieving the maximum score.

Internal consistency was calculated with Cronbach’s alpha for the first CHQ-D measurement of the participants of the validation cohort. Internal consistency as determined by Cronbach’s alpha is deemed acceptable when greater than 0.7 and excellent when greater than 0.8.

To estimate test-retest reliability, the intraclass correlation coefficient (ICC) was calculated. The ICC estimates and their 95% confidence intervals were calculated for the total CHQ score and each item of the CHQ based on a single-rating, absolute-agreement, 2-way mixed-effects model [10]. ICC values of less than 0.5 indicate poor reliability, values between 0.5 and 0.75 moderate reliability, values between 0.75 and 0.9 good reliability, and values greater than 0.90 excellent reliability [10].

To visualize the reproducibility and the degree of similarity between both completed questionnaires, a Bland Altman plot was created [11]. The 95% limits of agreement (LOA) is calculated as the mean difference between the two measurements of the total CHQ-D score ± 1.96 standard deviations.

All statistical analyses were performed with RStudio version 4. Two-tailed *p* values less than 0.05 were considered statistically significant.

## Results

### Translation

The questionnaire was divided into 41 items (title, 2 parts instruction, 31 questions, 7 options of choice) which were translated by the two independent translators. In 31.7% (*n* = 13) of the items, there was complete similarity between both preliminary translations; in 41.5% (*n* = 17), there was partial similarity; and in 26.8% (*n* = 11), there was minimal or no similarity. The minimal differences mostly consisted of a different sentence structure, and in case of no similarity, it was due to a different choice of words. After comparing and discussing the differences, consensus was achieved in all cases resulting in a preliminary version of the translated questionnaire. After comparing this preliminary version of the translation with the original CHQ questionnaire, the adjudicator had content-related comments on 3 items. In consultation with translators, reviewers, and adjudicator, 1 adjustment was made before completing the pre-final version of the questionnaire.

### Participants

**Fig. 2:**
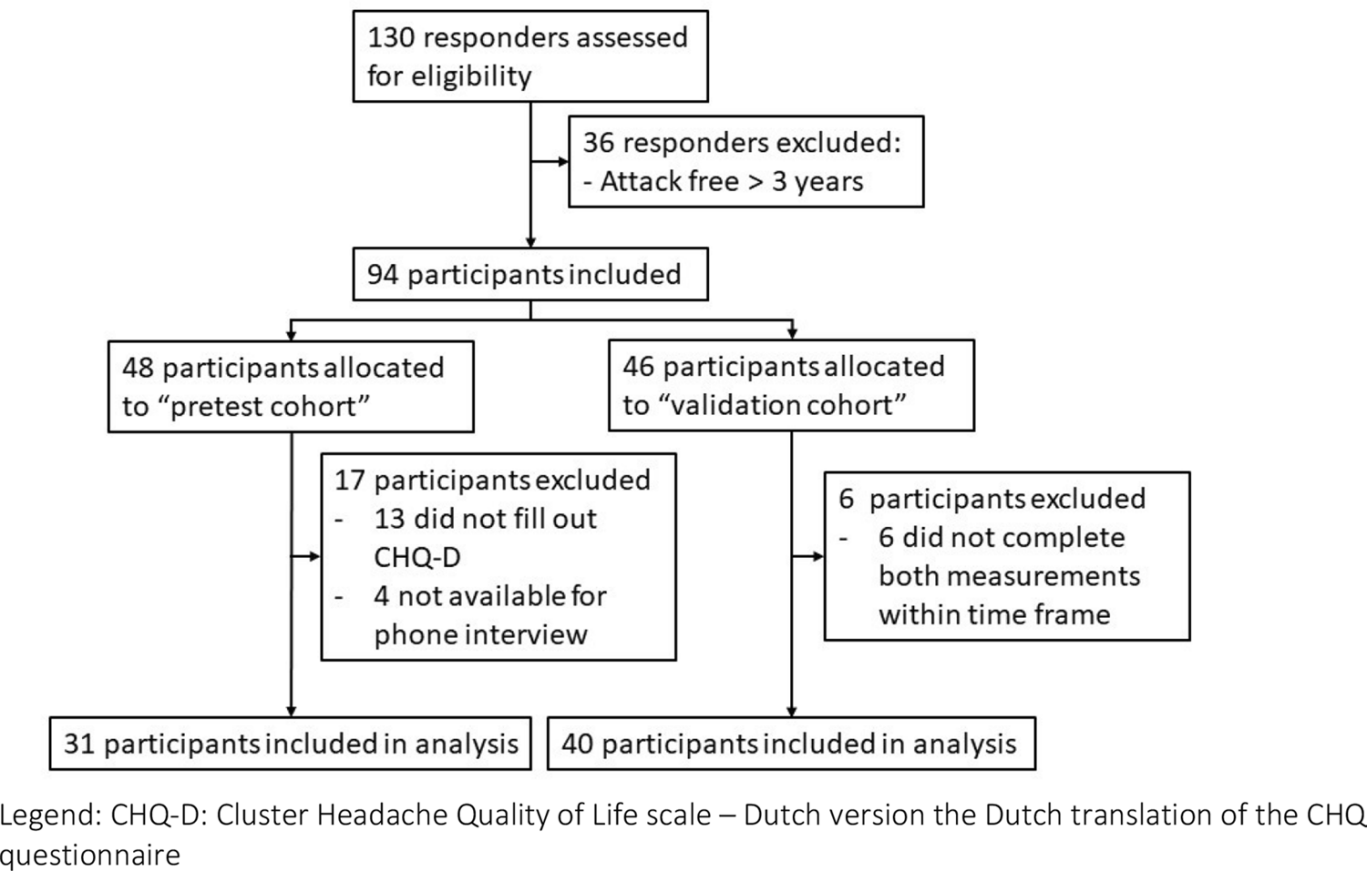
Flow chart. Legend: CHQ-D, Cluster Headache Quality of Life scale—Dutch version the Dutch translation of the CHQ questionnaire

Nine hundred individuals with cluster headache from the LUCA cohort were invited for participation. One hundred thirty of the 900 (14%) eligible persons with cluster headache from the LUCA cohort were interested, of whom thirty-six people (27.7%) were excluded because they were cluster headache attack–free for more than 3 years (Fig. 2). The final groups who participated in our study (*n* = 31 and *n* = 40) were a good representation of the total invited cohort (*n* = 900) for age, sex, and education. The final group was more often of the chronic cluster headache subtype than the total invited cohort (50.7% vs 27.7% cCH).

The pre-final version of the translated questionnaire was sent to 48 participants for preliminary testing, of whom 31 (64.4%) completed the questionnaire and had an interview by phone. The revised and final CHQ-D (Supplemental 1) was sent to 46 participants for validation, of which 40 (87.0%) completed both measurements of the questionnaire. The demographic and clinical characteristics did not differ between the test cohort and the validation cohort (Supplemental 2).

### Preliminary testing

The complete results of the preliminary tests are shown in Table 1. Participants took a median time of 10 min (IQR: 10.0, 17.5) to complete the questionnaire, with 90% finishing it within 20 min. The majority of participants (74.2%) did not find it burdensome at all, and none experienced it as “very burdensome.” Higher survey burden scores were mostly due to the fact that the questionnaire was found “challenging” regarding the severity of their condition.

**Table 1:** Results of the evaluation of the pre-final version the translation of the CHQ in the test cohort

Pre-final CHQ-D results	Test cohort (*N* = 31)
Duration completion questionnaire (min), median [IQR])	10.0 [10.0, 17.5]
Burdening completing questionnaire*, *N* (%)	
1	23 (74.2)
2	5 (16.1)
3	2 ( 6.5)
4	1 ( 3.2)
5	0
6	0
Total score CHQ-D, mean (± SD)	63.0 (16.1)
Subscores, mean (± SD)	
Restrictions of activities of daily living	24.0 (6.9)
Impact on mood and interpersonal relationships	20.3 (7.7)
Pain and anxiety	5.4 (1.7)
Lack of vitality	13.32 (3.60)
Self-reported satisfaction with life, median [IQR])	7.0 [6.0, 8.0]

Legend: *CHQ-D*, Cluster Headache Quality of Life scale—Dutch version of the Dutch translation of the CHQ questionnaire; *IQR*, interquartile range; *SD*, standard deviation

^*^This was scored using a 6-point Likert scale, where 1 was “not burdensome at all” and 6 “very burdensome”

To produce the CHQ-D, we made five small modifications based on pretesting. After completion of the questionnaire and evaluation by phone, 9 participants (29%) had minor remarks about the clarity or wording of the questionnaire. No significant correlation was found between level of education level and having comments (*p* = 0.95). In total, there were 24 remarks divided over 10 items of the questionnaire. Most of the comments focused on the common part of the question “Hoe vaak heeft u/bent u vanwege uw clusterhoofdpijn in de afgelopen maand of tijdens uw laatste episode:” (in English “Due to cluster headache, in the past month or last episode, how often have you …”), questions 1, 2, and 3, as depicted in Supplemental 3.

After a review of all comments by the translators and the reviewer, five adjustments were made to the CHQ-D. The remaining suggestions were not incorporated in the final translation because they would either change the question in a way that did not match the original question or were aimed at the overall content and not the linguistics of the questionnaire (e.g., missing some elements of QoL in the questionnaire).

### Validation

No floor or ceiling effect was observed as none of the participants scored the minimum (0) or maximum (112) score (range 22 to 99).

The mean time between completing the two measurements was 2.08 days (SD ± 0.27). Three participants completed both measurements on day 1 and day 4 with an interval of 3 instead of the intended 2 days. The reliability of the CHQ-D was excellent with a Cronbach’s alpha of 0.94 for the first measurement of the CHQ-D-questionnaire (Table 2). Table 2 shows the Cronbach’s alpha for each of the four subcategories. In addition, the corrected item to total correlation is depicted for items in their respective subcategory.

**Table 2:** Cronbach’s alpha for total CHQ-D score and underlying subscales in the validation cohort

Item	No. items	Cronbach’s alpha
Total score CHQ-D	28	0.94
Subscales		
Restrictions of activities of daily living	9	0.93
Impact on mood and interpersonal relationships	12	0.88
Pain and anxiety	2	0.61
Lack of vitality	5	0.75

Legend: *CHQ-D*, Cluster Headache Quality of Life scale—Dutch version; *IQR*, interquartile range; *SD*, standard deviation

**Fig. 3:**
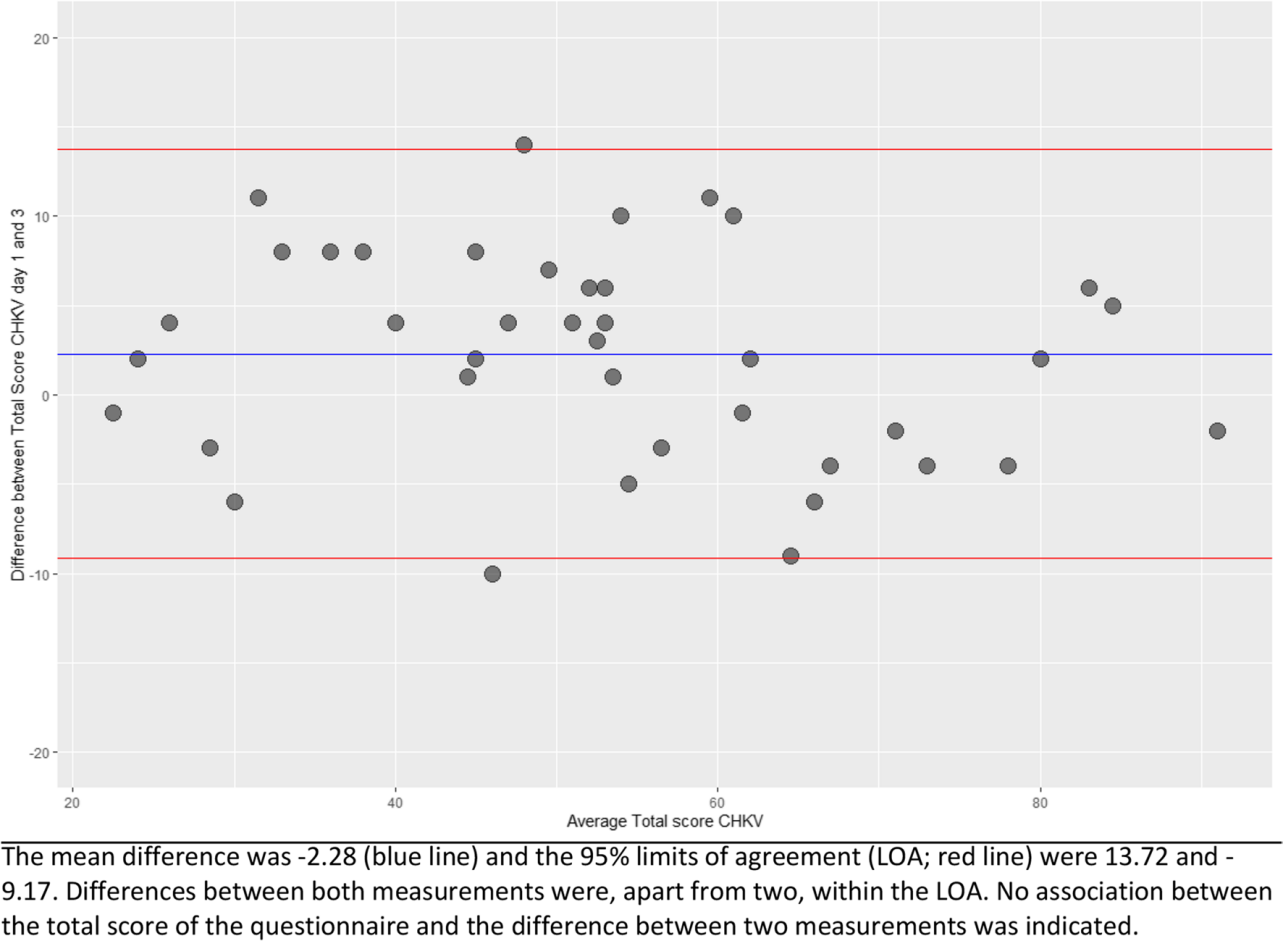
Bland Altman plot of the difference between the total CHQ-D scores for both measurements of the CHQ-D (completion *on day 1 vs day 3*)

The total score of the CHQ-D differed by − 2.3 between both measurements with a 95% limit of agreement (LOA) between − 9.2 and 13.7 (Table 3) as visualized in the Bland–Altman plot (Fig. 3). All but two (5%) participants were within the LOA. No relationship between the total score of the questionnaire and the difference between the two measurements was observed.

**Table 3:** Results final CHQ-D of the validation cohort

Item	Validation cohort (*N* = 40)		
	Time 1	Time 2	Difference
Total score CHQ-D, mean (± SD)	54.1 (17.0)	51.8 (18.10)	− 2.3 (5.8)
Subscores, mean (± SD)			
Restrictions of activities of daily living	21.3 (6.6)	19.9 (6.78)	− 1.5 (3.1)
Impact on mood and interpersonal relationships	15.8 (8.6)	15.4 (9.11)	− 0.4 (3.7)
Pain and anxiety	5.3 (1.6)	4.9 (1.42)	− 0.4 (0.9)
Lack of vitality	11.6 (3.1)	11.6 (3.53)	− 0.5 (2.0)
Self-reported satisfaction with life, median [IQR])	7.0 [6.0, 8.0]	7.0 [6.0, 8.0]	0.0 (0.0, 0.0)

Legend: *CHQ-D*, Cluster Headache Quality of Life scale—Dutch version; *IQR*, interquartile range; *SD*, standard deviation

The test–retest reliability for the complete CHQ-D-questionnaire was deemed excellent with an intraclass correlation coefficient (ICC) of 0.94 (95% CI 0.88; 0.97 (Table 4). All subscores had good reliability (ICC > 0.75). Each individual item had at least moderate reliability (ICC > 0.6), with more than half of the items having at least good reliability (ICC > 0.75) (Supplemental 5).

**Table 4:** Intraclass correlation coefficient for total CHQ-D score and underlying subscales of the validation cohort

Item	Intraclass correlation coefficient	95% CI	
		Lower bound	Upper bound
Total score CHQ-D	0.938	0.877	0.968
Subscales			
Restrictions of activities of daily living	0.875	0.742	0.937
Impact on mood and interpersonal relationships	0.914	0.844	0.954
Pain and anxiety	0.799	0.627	0.893
Lack of vitality	0.820	0.685	0.901
Overall satisfaction with life	0.896	0.812	0.944

Legend: *CHQ*, Cluster Headache Quality of Life scale; *CI*, confidence interval

## Discussion

This study describes the translation and validation of the CHQ from English to Dutch. The translation process with the TRAPD method included cross-cultural validation and resulted in the Dutch version of the CHQ. The reproducibility and internal consistency are good to excellent, and consistent with the validation of the original CHQ [3]. Due to the absence of floor and ceiling effects in our analyses, the CHQ-D is applicable to patients with a very low or a very high QoL as well.

The CHQ-D enables future studies to quantify different aspects of the QoL of the Dutch-speaking cluster headache population. Greater emphasis on PROMS in clinical trials demonstrates the need for better and more specific measures of quality of life. Moreover, determinants of QoL could identify unmet needs of individuals with cluster headache and highlight areas where more (non)-pharmacological interventions are indicated.

The CHQ is able to detect differences in impairment of QoL between mild and severe cluster headache [3]. This creates the possibility to incorporate this measure in longitudinal studies, correlating intra-patient variability of QoL to fluctuations in cluster headache severity (i.e.., attack frequency). More information should be gathered about factors that impact QoL (e.g., age/sex differences, treatment effects incl. adverse events) of the Dutch cluster headache population. Ultimately, increasing the QoL of people with cluster headache.

One of the strengths of this study is the use of the TRAPD translation guideline, which has been followed strictly [5]. This resulted in a translation that not only is grammatically correct in Dutch, but also includes linguistic nuances and cross-cultural differences as well. The accuracy and quality of the translation process were highlighted by the fact that the pre-final version of the translation hardly needed any changes after the pre-tests. The results can be generalized to the entire Dutch-speaking cluster headache population, since participants were included from the well-validated nationwide web-based LUCA cohort and were from different parts of the Netherlands with different Dutch dialects.

The contribution of chronic (45–51%) cluster headache patients is higher than expected based on the known prevalence of the chronic type in the general cluster headache population and our total invited cohort [12]. Since chronic cluster headache is correlated with a lower QoL [13], the use of QoL scales such as the CHQ-D is especially important for individuals with chronic cluster headache. The relative overrepresentation of chronic cluster headache in our study cohort therefore increases the practical validity of the CHQ-D. There might be an overestimation of the test-retest reliability due to the 2-day interval between the two measurements of the CHQ-D. The 2-day interval between the two measurements was intended to keep the disease activity and other possible confounding factors as stable as possible. Unfortunately, this relatively short interval could inadvertently have led to the recollection of answers from the first measurement. However, this possible overestimation is expected to be limited since a 2-day interval is considered to be equivalent to a 2-week interval [9].

In conclusion, the Dutch translation of the CHQ scale, the CHQ-D, is a valid, reliable, easy-to-use, and practical instrument to assess cluster headache–related disability and impairment on the QoL and is comparable to the original English version of the scale. The CHQ-D can be used in the clinical setting to monitor QoL as part of the regular patient care and aim to use CHQ-D as PROM in clinical research in the Netherlands to enforce international collaborations and comparisons of studies.

### Supplementary information

Below is the link to the electronic supplementary material.

Supplementary file1 (DOCX 21 KB)

Supplementary file2 (DOCX 17 KB)

Supplementary file3 (DOCX 17 KB)

Supplementary file4 (DOCX 30 KB)

Supplementary file5 (DOCX 25 KB)

## Data Availability

The data used for this study, including de-identified individual data and a data dictionary defining each field within the dataset, can be made available on a secure server by the corresponding author on reasonable request. These data will be made available only after full-text publication of the primary report.
