# Supplementary material for: Translating the Cluster Headache Quality of Life Questionnaire (CHQ) from English to Dutch with the TRAPD method
Source: Neurol Sci. 2023 Oct 6;45(3):1217–24. doi: 10.1007/s10072-023-07088-x (PMC10858103; doi:10.1007/s10072-023-07088-x)
Supplement: Supplementary file 1 — Supplementary file1 (DOCX 21 KB) [file 10072_2023_7088_MOESM1_ESM.docx]

**Supplemental 1. Final translation**

**CLUSTERHOOFDPIJN KWALITEIT VAN LEVEN VRAGENLIJST (CHQ-D)**

Hoe vaak heeft u in de afgelopen maand een clusterhoofdpijn aanval gehad? _________

Beantwoord de onderstaande vragen om aan te geven hoe vaak clusterhoofdpijn verschillende aspecten van uw leven heeft beïnvloed.

De vragen gaan over de afgelopen maand, tenzij u geen aanvallen heeft gehad. Dan gaat het over uw meest recente clusterhoofdpijn episode.

Kruis bij elke vraag slechts één vakje aan. Laat geen vragen onbeantwoord.

| **Vanwege uw clusterhoofdpijn, hoe vaak heeft u/bent u:** | Nooit | Zelden | Soms | Vaak | Altijd |
| --- | --- | --- | --- | --- | --- |
| 1. Vermeden om de deur uit te gaan |  |  |  |  |  |
| 1. Vermeden om plannen te maken vanwege de onvoorspelbaarheid van clusterhoofdpijn (bijv. vakanties) |  |  |  |  |  |
| 1. Zich niet in staat gevoeld om taken op het werk te voltooien |  |  |  |  |  |
| 1. Moeite gehad om activiteiten in uw vrije tijd te ondernemen (bijv. naar de bioscoop of het theater gaan, etc.) |  |  |  |  |  |
| 1. Drukke en rumoerige plekken vermeden (bijv. openbaar vervoer, kroegen, etc.) |  |  |  |  |  |
| 1. Het gevoel gehad dat de ernst van uw clusterhoofdpijn uw dagelijks leven heeft beïnvloed |  |  |  |  |  |
| 1. Minder betrokken geweest bij familiegelegenheden (bijv. omgang met kinderen, het plannen van vakanties, etc.) |  |  |  |  |  |
| 1. Niet in staat geweest om tijd te besteden aan sociale activiteiten/om te gaan met familie en vrienden |  |  |  |  |  |
| 1. Niet in staat geweest om dagelijkse doelen te behalen en dagelijkse bezigheden en taken uit te voeren |  |  |  |  |  |
| 1. Zich minder gerespecteerd gevoeld door anderen |  |  |  |  |  |
| 1. Problemen gehad met hechte persoonlijke relaties |  |  |  |  |  |
| 1. Het gevoel gehad tot last te zijn voor familie en vrienden |  |  |  |  |  |
| 1. Een zelfbewust of ongemakkelijk gevoel gehad over uw uiterlijk na een clusterhoofdpijnaanval (bijv. door gezwollen/rode ogen of een bezweet gezicht, etc.) |  |  |  |  |  |
| 1. Het gevoel gehad dat anderen uw clusterhoofdpijn niet serieus namen |  |  |  |  |  |
| 1. Zich agressief gevoeld |  |  |  |  |  |
| 1. Een slecht gevoel gehad over uzelf, minder zelfvertrouwen ervaren of zich onbelangrijk gevoeld |  |  |  |  |  |
| 1. Zichzelf iets willen aandoen of suïcidale gedachten gehad |  |  |  |  |  |
| 1. Prikkelbaar, ongeduldig of minder verdraagzaam geweest |  |  |  |  |  |
| 1. Vergeetachtig geweest (bijv. afspraken gemist) |  |  |  |  |  |
| 1. Niet in staat geweest om voor uw uiterlijk te zorgen (bijv. douchen, make-up opdoen, omkleden, etc.) |  |  |  |  |  |
| 1. Zich geïsoleerd, eenzaam of kwetsbaar gevoeld |  |  |  |  |  |
| 1. Het gevoel gehad dat uw pijn ondraaglijk was als deze niet behandeld zou worden |  |  |  |  |  |
| 1. Gevreesd dat uw hoofdpijn niet over zou gaan |  |  |  |  |  |
| 1. Een gebrek aan energie gehad en zich constant moe gevoeld |  |  |  |  |  |
| 1. Zich slaperig, uitgeput of niet goed in staat gevoeld om te concentreren door nachtelijke aanvallen van clusterhoofdpijn |  |  |  |  |  |
| 1. Concentratieproblemen gehad (bijv. bij krant lezen of tv kijken, etc.) |  |  |  |  |  |
| 1. Zich niet in staat gevoeld helder na te denken |  |  |  |  |  |
| 1. Zich gespannen of angstig gevoeld |  |  |  |  |  |

Hoe tevreden bent u met uw leven op een schaal van 1-10? ______

(1 = helemaal niet tevreden, 10 = zeer tevreden) ­­
