# Supplementary material for: Translating the Cluster Headache Quality of Life Questionnaire (CHQ) from English to Dutch with the TRAPD method
Source: Neurol Sci. 2023 Oct 6;45(3):1217–24. doi: 10.1007/s10072-023-07088-x (PMC10858103; doi:10.1007/s10072-023-07088-x)
Supplement: Supplementary file 2 — Supplementary file2 (DOCX 17 KB) [file 10072_2023_7088_MOESM2_ESM.docx]

**Supplemental 2.** Demographics of cluster headache population of the Test and Validation phase.

|  | Test cohort (n=31) | Validation cohort (n=40) |
| --- | --- | --- |
| **Demographic characteristics** |  |  |
| Male, N (%) | 17 (54.8) | 25 (62.5) |
| Age (years), median [IQR] | 59.0 [53.0, 63.0] | 54.5[41.8, 63.3] |
| Education, N (%): |  |  |
| - Primary education | 0 ( 0.0) | 1 ( 2.5) |
| - Secondary education | 4 (12.9) | 5 (12.5) |
| - Secondary vocational education | 9 (29.0) | 14 (35.0) |
| - Higher professional education | 13 (41.9) | 13 (32.5) |
| - University education | 5 (16.1) | 7 (17.5) |
| Episodic CH, N (%) | 17 (54.8) | 19 (47.5) |
| In-episode, N (%) | 2 (11.8) | 2 (12.5) |
| No CH attacks last month, N [IQR] | 17.5 [2.5, 28.5] | 15.0 [5.0,57.5] |

Legend: IQR: Interquartile Range, CH: Cluster Headache
