# Supplementary material for: Translating the Cluster Headache Quality of Life Questionnaire (CHQ) from English to Dutch with the TRAPD method
Source: Neurol Sci. 2023 Oct 6;45(3):1217–24. doi: 10.1007/s10072-023-07088-x (PMC10858103; doi:10.1007/s10072-023-07088-x)
Supplement: Supplementary file 3 — Supplementary file3 (DOCX 17 KB) [file 10072_2023_7088_MOESM3_ESM.docx]

**Supplemental 3**: Most commented items of the pre-final version of the translation of the CHQ after pretesting

| **Item** | **Number of comments, N (% of all respondents)** |
| --- | --- |
| Preliminary Question: *‘Hoe vaak heeft u/bent u vanwege uw clusterhoofdpijn in de afgelopen maand of tijdens uw laatste episode’ (translated in English: “Due to cluster headache, in the past month or last episode, how often have you:)”* | 6 (19.4) |
| Question 1: *“Het vermeden om de deur uit te gaan” (translated in English: “Avoided leaving the house”)* | 4 (12.9) |
| Question 2: *“Het vermeden om plannen te maken vanwege de onvoorspelbaarheid van clusterhoofdpijn” (translated in English: “Avoided making plans due to unpredictability of cluster headache”* | 4 (12.9) |
| Question 3: *“Zich niet in staat gevoeld om taken op werk te voltooien” (translated in English “Felt unable to complete duties at work”)* | 3 ( 9.7) |
| Final Question: *“Beoordeel op de onderstaande schaal uw algehele tevredenheid over uw leven met een markering op een bij u passend punt*.” *(translated in English: Please rate your overall satisfaction with your life by placing a vertical line on the scale below at an appropriate point.”)* | 2 (6.5) |
