# Supplementary material for: Translating the Cluster Headache Quality of Life Questionnaire (CHQ) from English to Dutch with the TRAPD method
Source: Neurol Sci. 2023 Oct 6;45(3):1217–24. doi: 10.1007/s10072-023-07088-x (PMC10858103; doi:10.1007/s10072-023-07088-x)
Supplement: Supplementary file 4 — Supplementary file4 (DOCX 30 KB) [file 10072_2023_7088_MOESM4_ESM.docx]

**Supplemental 4**. English original Cluster headache quality of life questionnaire (CHQ)

CLUSTER HEADACHE QUALITY OF LIFE QUESTIONNAIRE (CHQ)

How many times have you experienced a cluster headache attack during the last month?

Please complete the following items to indicate how often cluster headache has affected various aspects of your life DURING THE LAST MONTH or DURING YOUR MOST RECENT CLUSTER HEADACHE EPISODE

Please tick only one box for each item. Do not leave any item blank.

| **Due to cluster headache, in the past month or last episode, how often**  **have you:** | **Never** | **Occasionally** | **Sometimes** | **Often** | **Always** |
| --- | --- | --- | --- | --- | --- |
| 1. Avoided leaving the house |  |  |  |  |  |
| 2. Avoided making plans due to unpredictability of cluster headache  e.g. holidays |  |  |  |  |  |
| 3. Felt unable to complete duties at work |  |  |  |  |  |
| 4. Had difficulty in getting involved in  leisure activities e.g. cinema, theatre, etc? |  |  |  |  |  |
| 5. Avoided crowded and noisy places  e.g. public transport, pubs, etc |  |  |  |  |  |
| 6. Felt that the severity of cluster  headache affected your daily activities |  |  |  |  |  |
| 7. Been less involved in family affairs  e.g. interaction with children, planning holidays |  |  |  |  |  |
| 8. Been unable to socialise/spend time with friends and family |  |  |  |  |  |
| 9. Been unable to achieve your daily  goals and carry out routines and chores |  |  |  |  |  |
| 10. Felt less respected by others |  |  |  |  |  |
| 11. Had problems with close personal  relationship |  |  |  |  |  |
| 12. Felt you were a burden on family and friends |  |  |  |  |  |
| 13. Felt self-conscious and uncomfortable  about your appearance after a cluster headache attack (eg swelling/redness of eyes and facial sweating, etc) |  |  |  |  |  |
| 14. Felt that others are dismissive of your  cluster headaches |  |  |  |  |  |
| 15. Felt aggressive |  |  |  |  |  |
| 16. Felt bad about yourself, lost self-  confidence or felt worthless |  |  |  |  |  |
| **Due to cluster headache, in the past month or last episode, how often have you:** | **Never** | **Occasionally** | **Sometimes** | **Often** | **Always** |
| 17. Felt like harming yourself or suicidal |  |  |  |  |  |
| 18. Been irritable, impatient or less  tolerant |  |  |  |  |  |
| 19. Been forgetful e.g. missed appointments |  |  |  |  |  |
| 20. Been unable to take care of your  appearance (eg take a bath, put make- up on, change clothes, etc) |  |  |  |  |  |
| 21. Felt isolated, lonely or vulnerable |  |  |  |  |  |
| 22. Found your pain is unbearable if  untreated |  |  |  |  |  |
| 23. Dreaded that the headache would not go away |  |  |  |  |  |
| 24. Felt lacking in energy and constantly  tired |  |  |  |  |  |
| 25. Felt sleepy, worn out or less able to concentrate due to nocturnal attacks  of cluster headache |  |  |  |  |  |
| 26. Had problems concentrating e.g. reading paper, watching TV, etc |  |  |  |  |  |
| 27. Been unable to think clearly |  |  |  |  |  |
| 28. Felt tense or anxious |  |  |  |  |  |

Please rate your overall satisfaction with your life by placing a vertical line on the scale below at an appropriate point

| |

Not at all satisfied Very satisfied
