# Supplementary material for: Translating the Cluster Headache Quality of Life Questionnaire (CHQ) from English to Dutch with the TRAPD method
Source: Neurol Sci. 2023 Oct 6;45(3):1217–24. doi: 10.1007/s10072-023-07088-x (PMC10858103; doi:10.1007/s10072-023-07088-x)
Supplement: Supplementary file 5 — Supplementary file5 (DOCX 25 KB) [file 10072_2023_7088_MOESM5_ESM.docx]

**Supplemental 5**. Intraclass Correlation Coefficient for all individual questions

| Item | Intraclass correlation coefficient | 95% CI | |  | F Test With True Value 0 | | | |
| --- | --- | --- | --- | --- | --- | --- | --- | --- |
|  |  | Lower bound | Upper bound |  | Value | *df*1 | *df2* | Sig |
| Restriction of ADL |  |  |  |  |  |  |  |  |
| 1. Avoided leaving the house | 0.755 | 0.581 | 0.862 |  | 6.99 | 39 | 39 | 8.4e-09 |
| 2. Avoided making plans due to unpredictability of cluster headache e.g. holidays | 0.83 | 0.703 | 0.906 |  | 10.7 | 39 | 39.1 | 6.89e-12 |
| 3. Felt unable to complete duties at work | 0.618 | 0.374 | 0.78 |  | 4.61 | 39 | 32.5 | 1.18e-05 |
| 4. Had difficulty in getting involved in leisure activities e.g. cinema, theatre, etc | 0.709 | 0.513 | 0.835 |  | 5.78 | 39 | 39.3 | 1.21e-07 |
| 5. Avoided crowded and noisy places e.g. public transport, pubs, etc | 0.744 | 0.566 | 0.856 |  | 7.07 | 39 | 38.4 | 8.84e-09 |
| 6. Felt that the severity of cluster headache affected your daily activities | 0.688 | 0.483 | 0.821 |  | 5.63 | 39 | 38.2 | 2.42e-07 |
| 7. Been less involved in family affairs e.g. interaction with children, planning holidays | 0.742 | 0.563 | 0.854 |  | 6.99 | 39 | 38.6 | 9.74e-09 |
| 8. Been unable to socialise/spend time with friends and family | 0.636 | 0.39 | 0.793 |  | 5 | 39 | 29.2 | 1.11e-05 |
| 9. Been unable to achieve your daily goals and carry out routines and chores | 0.681 | 0.475 | 0.817 |  | 5.43 | 39 | 39.2 | 3.04e-07 |
| Impact on mood and interpersonal relationships |  |  |  |  |  |  |  |  |
| 10. Felt less respected by others | 0.834 | 0.695 | 0.91 |  | 12.1 | 39 | 31 | 1.23e-10 |
| 11. Had problems with close personal relationship | 0.843 | 0.723 | 0.914 |  | 11.5 | 39 | 39 | 3.33e-12 |
| 12. Felt you were a burden on family and friends | 0.736 | 0.549 | 0.852 |  | 7.03 | 39 | 35 | 3e-08 |
| 13. Felt self-conscious and uncomfortable about your appearance after a cluster headache attack (e.g. swelling/redness of eyes and facial sweating, etc) | 0.829 | 0.701 | 0.906 |  | 10.6 | 39 | 39.7 | 9.16e-12 |
| 14. Felt that others are dismissive of your cluster headaches | 0.799 | 0.653 | 0.888 |  | 9.08 | 39 | 39.8 | 1.1e-10 |
| 15. Felt aggressive | 0.911 | 0.838 | 0.952 |  | 21 | 39 | 39.2 | 8.59e-17 |
| 16. Felt bad about yourself, lost self-confidence or felt worthless | 0.76 | 0.59 | 0.865 |  | 7.22 | 39 | 39.5 | 4.41e-09 |
| 17. Felt like harming yourself or suicidal | 0.736 | 0.554 | 0.851 |  | 6.5 | 39 | 39.6 | 2.05e-08 |
| 18. Been irritable, impatient or less tolerant | 0.706 | 0.508 | 0.833 |  | 5.72 | 39 | 39.5 | 1.32e-07 |
| 19. Been forgetful e.g. missed appointments | 0.729 | 0.54 | 0.847 |  | 6.76 | 39 | 36.1 | 3.56e-08 |
| 20. Been unable to take care of your appearance (e.g. take a bath, put make-up on, change clothes, etc) | 0.689 | 0.482 | 0.823 |  | 5.33 | 39 | 39.1 | 4.07e-07 |
| 21. Felt isolated, lonely or vulnerable | 0.719 | 0.527 | 0.841 |  | 6 | 39 | 39 | 7.93e-08 |
| Pain and anxiety |  |  |  |  |  |  |  |  |
| 22. Found your pain is unbearable if untreated | 0.757 | 0.586 | 0.863 |  | 7.16 | 39 | 39.8 | 4.58e-09 |
| 23. Dreaded that the headache would not go away | 0.685 | 0.45 | 0.826 |  | 6.07 | 39 | 26.5 | 3.42e-06 |
| Lack of vitality |  |  |  |  |  |  |  |  |
| 24. Felt lacking in energy and constantly tired | 0.748 | 0.57 | 0.858 |  | 7.24 | 39 | 37.6 | 8.17e-09 |
| 25. Felt sleepy, worn out or less able to concentrate due to nocturnal attacks of cluster headache | 0.701 | 0.503 | 0.83 |  | 5.66 | 39 | 39.8 | 1.46e-07 |
| 26. Had problems concentrating e.g. reading paper, watching TV, etc | 0.733 | 0.549 | 0.849 |  | 6.41 | 39 | 39.6 | 2.49e-08 |
| 27. Been unable to think clearly | 0.715 | 0.522 | 0.838 |  | 5.95 | 39 | 39.7 | 7.33e-08 |
| 28. Felt tense or anxious | 0.653 | 0.434 | 0.799 |  | 4.93 | 39 | 38.7 | 1.25e-06 |
| How many times have you experienced a cluster headache attack during the last month? | 0.993 | 0.987 | 0.996 |  | 290 | 39 | 39.7 | 7.23e-39 |
| Please rate your overall satisfaction with your life by placing a vertical line on the scale below at an appropriate point | 0.896 | 0.812 | 0.944 |  | 17.9 | 39 | 39.5 | 1.23e-15 |
